# In Vivo Hepatoprotective Effects of a Peptide Fraction from Krill Protein Hydrolysates against Alcohol-Induced Oxidative Damage

**DOI:** 10.3390/md17120690

**Published:** 2019-12-07

**Authors:** Soo Yeon Park, Ilekuttige Priyan Shanura Fernando, Eui Joeng Han, Min Ju Kim, Kyungsook Jung, Dong-Soo Kang, Chang-Bum Ahn, Ginnae Ahn

**Affiliations:** 1 Department of Marine Bio-Food Sciences, Chonnam National University, Yeosu 59626, Korea; soo6683@naver.com (S.Y.P.); shanurabru@gmail.com (I.P.S.F.); dskang@jnu.ac.kr (D.-S.K.); 2 Department of Food Technology and Nutrition, Chonnam National University, Yeosu 59626, Korea; iosu5772@naver.com (E.J.H.); mijoo92@naver.com (M.J.K.); 9321@jnu.ac.kr (C.-B.A.); 3 Biomaterials Research Center, Korea Research Institute of Bioscience and Biotechnology (KRIBB), 181 Ipsin-gil, Jeongeup-si, Jeonbuk 56212, Korea; jungks@kribb.re.kr

**Keywords:** alcoholic liver disease, *Euphausia superba*, protein hydrolysate, antioxidant peptides, ultrafiltration, glutamic oxaloacetic transaminase, glutamic pyruvic transaminase

## Abstract

Background: Krill (*Euphausia superba*) represent the largest animal biomass on earth, and are a rich source of high-quality protein with essential amino acids. Krill-derived peptides are renowned for their antioxidant activities. Hence, these peptides may have protective effects against oxidative stress. Alcoholic liver disease is a prevalent cause of death worldwide. The present study explores the hepatoprotective effects of krill peptide hydrolysate fractions against ethanol-induced liver damage in BALB/c mice. Methods: Hydrolysis was carried out by mimicking the gastrointestinal digestion environment and the filtrate was fractionated based on molecular weight (<1 kDa, 1–3 kDa, and >3 kDa). The 1–3 kDa fraction (KPF), which indicated the highest antioxidant effect, was further investigated for its effect on weight and survival rate increase in mice and its influence on serum glutamic oxaloacetic transaminase, glutamic pyruvic transaminase, and liver cholesterol levels. Moreover, superoxide dismutase (SOD), catalase (CAT), and glutathione peroxidase (GPx) levels were measured, followed by Nrf2 and HO-1 expression. Histopathology studies were conducted to assess hepatic tissue damage. Results: KPF enhanced the weight and survival rate of mice while reducing serum glutamic oxaloacetic transaminase, glutamic pyruvic transaminase, and liver cholesterol levels. Moreover, KPF upregulated SOD, CAT, and GPx in liver tissues, while downregulating tumor necrosis factor α and interleukin-6 mRNA expression. KPF further increased Nrf2 and HO-1 expression and suppressed ethanol-induced apoptotic proteins in the liver. Histopathology of KPF-treated mice showed less hepatic tissue damage compared to ethanol-treated mice. Conclusions: Hydrolysates and bioactive peptides prepared from krill can be employed as functional foods to enhance liver function and health. Further investigations of KPF could lead to the development of functional foods.

## 1. Introduction

Long-term and heavy consumption of alcohol leads to hepatotoxicity, thus increasing the risk of chronic liver damage [1]. The pathogenesis of alcoholic liver disease (ALD) is a result of chronic alcohol usage. In the United States, approximately 44% of the 26,000 reported deaths due to cirrhosis are due to ALD [2]. In South Korea, next to Hepatitis B and C, 20% of chronic liver diseases are due to ALD [3]. Chronic alcohol consumption is associated with progressive liver disease ranging from steatosis to inflammation, development of hepatic cirrhosis, and the subsequent increase in the risk of hepatocellular carcinoma.

Alcohol is readily absorbed from the gastrointestinal tract, rapidly circulated, and gets uniformly distributed throughout the body. Metabolism of alcohol mainly takes place in the liver via oxidative enzymatic pathways involving enzymes such as alcohol dehydrogenase (ADH), catalase (CAT), and the microsomal ethanol oxidation system (MEOS). Free radicals are generated extensively in each pathway, altering cellular redox homeostasis [4]. ADH breaks alcohol down to acetaldehyde, whereas aldehyde dehydrogenase (ALDH) oxidizes acetaldehyde to acetate. The acetate is unstable and breaks down into water and carbon dioxide. MEOS is an auxiliary pathway that becomes active upon chronic alcohol induction. The pathway is catalyzed by cytochrome P450 enzymes. The isoform 2E1 of the cytochrome P450 (CYP2E1) system gets induced during chronic alcohol consumption. Activated CYP2E1 leads to increased ROS generation, including superoxide anions and hydroxyl radicals resulting in oxidative stress and cell death [5,6]. Moreover, peroxisomal activity in the liver contributes to ethanol oxidation, whereas ethanol reacts with H_2_O_2_ catalyzed by CAT, producing acetaldehyde and water. This mechanism might be more prominent in heavy ethanol consumers where there is an accumulation of fatty acids in the liver, due to the increased peroxisomal oxidation of fatty acids [4]. Additionally, ADH oxidation of ethanol and subsequent catabolism of acetaldehyde results in an increase in the NADH/NAD^+^ ratio in the cytoplasm and mitochondria. Increased NADH inhibits mitochondrial β-oxidation reactions inhibiting fatty acid catabolism, which causes the accumulation of intracellular lipids. ROS generated during alcohol metabolism not only increases the fat accumulation in hepatocytes, but also sensitizes the liver to subsequent cytokine outbreaks [5,7].

Given the context, antioxidant therapy is of interest as a potential treatment strategy for ALD. Krill peptides are renowned for their strong antioxidant effects and may have potential activity against oxidative stress [8,9]. Also, it has been reported that krill-derived peptides with molecular weights of 300–1400 Da reduce ice crystal formation during frozen storage in lizardfish myofibrils, leading to structural stabilization while inactivating Ca^2+^-ATPase activity. The present study is a continuation of our previous research, which supports the antioxidant activities of ultrafiltration fractions of krill (*Euphausia superba*) protein hydrolysates [9]. Based on our previous study, the 1–3 kDa fraction exhibited the highest antioxidant activity with a higher content of glutamic acid, aspartic acid, leucine, lysine, and arginine, which may have contributed to its effects. The current study was undertaken to prove the hypothesis that antioxidant peptides in krill protein hydrolysates (KPH) would exhibit protective effects against ethanol-induced oxidative damage in BALB/c mice.

## 2. Results

### 2.1. The 1–3 kDa Fraction (KPF) Increased Body Weight and Survival Rate in Ethanol-Induced Mice

**Figure 1:**
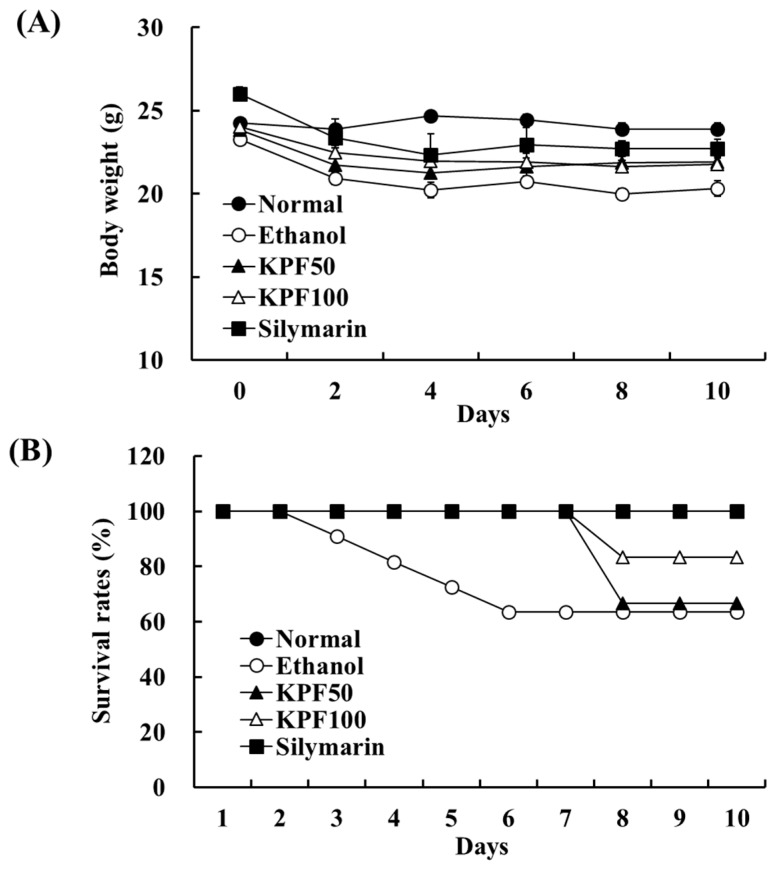
Changes in the body weight and survival rate of mice after KPF (1–3 kDa fraction of krill peptide hydrolysate) treatment. (**A**) Body weight gain in mice fed experimental diets for ten days. (**B**) Survival rates in experimental mice. Values: mean ± SE of three determinations (*n* = 6).

The body weight of the mice was monitored every two days during the experimental period. The changes in body weight in the mice are shown in Figure 1A. The final body weight gain in the ethanol group (20.33 ± 0.45 g) was significantly lower than that of the normal group (23.91 ± 0.35 g). The body weight gain in the KPF (50 and 100 mg/kg mice) groups was 21.92 ± 0.22 g and 21.77 ± 0.06 g, respectively. The survival rates of the experimental mice were monitored daily. As shown in Figure 1B, the survival rate in the control and silymarin groups was 100% during the trial period, whereas the survival rate in the ethanol group was 63.6%. In the case of the KPF (50 and 100 mg/kg mice) groups, the survival rate was 66.7% and 83.3%, respectively.

### 2.2. KPF Ameliorated Hepatic Biomarkers in Ethanol-Induced Mice

The influence of KPF on serum hepatic marker enzymes in ethanol-induced liver injured mice were investigated. As indicated in Table 1, the levels of AST and ALT were increased in the ethanol-treated group (92.88 ± 4.96 and 24.36 ± 1.21 IU/L) compared to those in the control group, indicating the weakening of liver function in the ethanol-treated group. However, the levels of AST and ALT were significantly (*p* < 0.05) decreased in mice administrated with different concentrations of KPF. The total serum cholesterol levels in mice after ten days of administration with experimental diets are shown in Table 1. KPF treatment significantly reduced (*p* < 0.05) the total cholesterol level in serum compared to those of the ethanol group. At a KPF concentration of 100 mg/kg, the total cholesterol level was reduced, reaching a level close to that of the control group and showing no significant difference. However, the activity of KPF at 100 mg/kg was not as strong as that of silymarin when considering all AST, ALT, and total cholesterol levels.

### 2.3. KPF Decreased Ethanol-Induced Lipid Peroxidation in Mice

**Figure 2:**
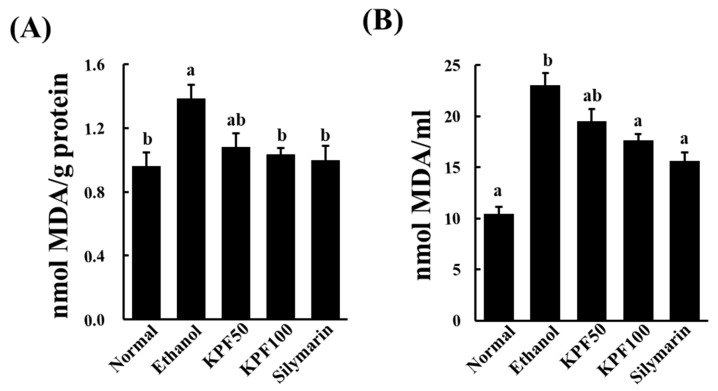
Changes in lipid peroxidation upon KPF treatment in ethanol-induced mice. Effect of KPF on the liver (**A**) and serum (**B**) MDA levels. Values: mean ± SE of three determinations (*n* = 6). Bars with different letters are significantly different (*p* < 0.05).

MDA levels in liver homogenates and serum were examined to evaluate the protective effect of KPF against lipid peroxidation. The MDA level in the liver homogenates of the ethanol (only)-treated group drastically increased compared to the control group, while MDA levels were significantly decreased in the KPF and silymarin-treated groups (Figure 2A). A similar trend was seen for the serum MDA levels (Figure 2B).

### 2.4. KPF Ameliorated Apoptosis-Related Protein Levels in Ethanol-Induced Mice

**Figure 3:**
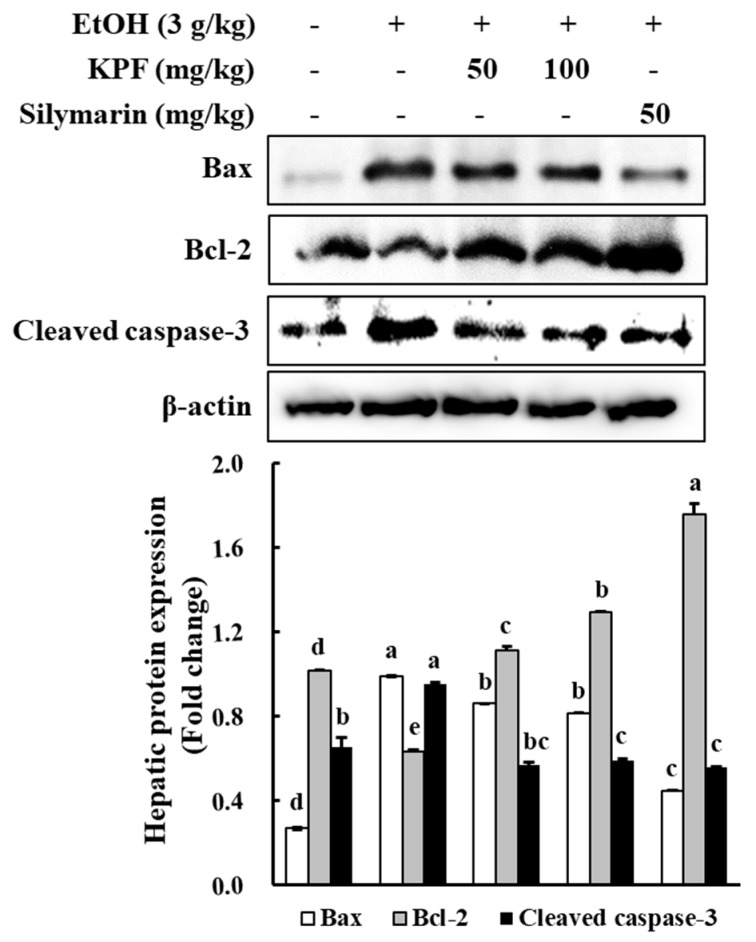
Effects of KPF on apoptosis-related protein expressions. Expression levels of cleaved caspase-3, Bcl-2, and Bax proteins in the liver tissue were analyzed by western blot analysis. Values: mean ± SE of three determinations (*n* = 6). Bars with different letters are significantly different (*p* < 0.05).

As indicated in Figure 3, alcohol administration increased the Bax levels but downregulated Bcl-2 expression, as compared to the control group. However, KPF administration resulted in a dose-dependent increase in Bcl-2 expression and a decrease in the expression of Bax. The fluctuation in cleaved caspase-3 levels was similar to that of Bax, where alcohol administration increased the cleaved caspase-3 level, whereas it was significantly decreased with dose-dependent administration of KPF.

### 2.5. KPF Enhanced Hepatic Antioxidant Enzymatic Defense in Ethanol-Induced Mice

**Figure 4:**
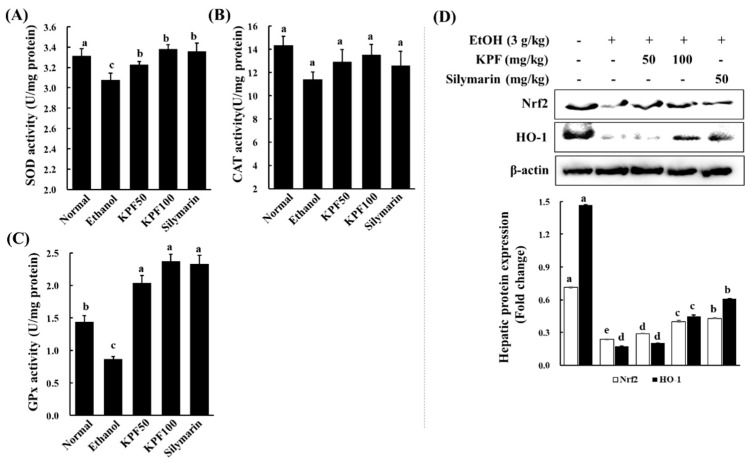
Effects of KPF on antioxidant enzyme levels in ethanol-induced mice. Determination of antioxidant enzyme activities (**A**) superoxide dismutase (SOD), (**B**) catalase (CAT) and (**C**) Glutathione peroxidase (GPx) in liver homogenates. (**D**) Effects of KPF on the hepatic levels of Nrf2 and HO-1 protein expression. Values: mean ± SE of three determinations (*n* = 6). Bars with different letters are significantly different (*p* < 0.05).

To evaluate the impact of KPF to prevent ethanol-induced hepatic damage, SOD, CAT and, GPx activities were measured in liver homogenates. Figure 4A–C shows the hepatic SOD, CAT, and GPx enzyme activities in the mice following ethanol and KPF administration. Hepatic SOD, CAT, and GPx levels in the ethanol group decreased significantly when compared with those in the control group. However, KPF-treated groups (50 and 100 mg/kg mice) showed significantly increased hepatic SOD and GPx activities due to ethanol-induced hepatic damage when compared with those in the ethanol group. High KPF dose also increased CAT activity when compared with the ethanol-treated group. To understand the underlying mechanism of the protective effects exerted by KPF on ethanol-induced oxidative stress, the protein expression of Nrf2 and HO-1, which are antioxidant-related genes, was examined by western blot analysis. As shown in Figure 4D, the decreased expression of HO-1 protein was observed in the ethanol group, and the protein expression of Nrf2 was also significantly downregulated by ethanol intake. However, KPF (100 mg/kg) or silymarin treatment significantly increased the expression of Nrf2 and HO-1.

### 2.6. KPF Ameliorated Hepatic Inflammation in Ethanol-Induced Mice

**Figure 5:**
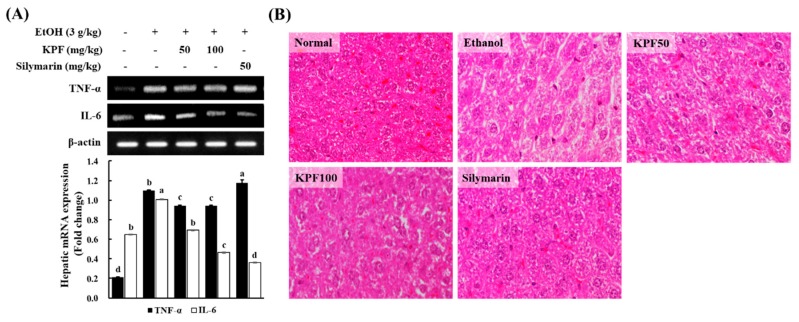
Effect of KPF on the hepatic levels of inflammatory cytokines in alcohol-induced mice. (**A**) RT-PCR analysis for mRNA levels of hepatic inflammatory cytokines. (**B**) Histopathological changes of mouse liver. Values: mean ± SE of three determinations (*n* = 6). Bars with different letters are significantly different (*p* < 0.05).

RT-PCR was implemented to evaluate the ethanol-induced expression of tumor necrosis factor (TNF)-α and interleukin (IL)-6 in the mouse liver. As shown in Figure 5A, alcohol treatment significantly increased the hepatic expression of TNF-α and IL-6 in mice compared to those in the control group. However, KPF treatment significantly lowered the inflammatory cytokine levels. The intake of alcohol led to liver injury, as indicated by hepatic histopathological changes. As shown in Figure 5B, the presence of slight micro-vesicular steatosis was observed in the ethanol group. However, KPF and silymarin treatment inhibited the alcohol-induced hepatic pathological changes. These data demonstrate that KPF could protect against ethanol-induced liver injury.

## 3. Discussion

Alcohol metabolism is linked to ROS production and oxidative stress, which involve both mitochondrial and microsomal systems. Ethanol causes the depletion of GSH and the levels of other antioxidant enzymes and decreases antioxidant cellular defense. It elevates malondialdehyde (MDA), hydroxynonenal (HNE), and hydroxyethyl radical (HER) protein adducts. These events result in cell death and tissue damage [4]. Dietary proteins are well recognized for their nutritional quality. However, their bioactivities have not received proper recognition, and many are unaware of such values. Recent research has shown that peptides from food proteins have desirable bioactivities in the human body. Bioactive peptides derived from marine proteins by enzymatic hydrolysis possess a wide range of bioactivities and have the potential to be used as ingredients in functional foods [10]. Krill, among other animal species, holds the record for having the largest biomass on earth [11]. It is a rich source of protein with essential amino acids and minerals. Though krill is abundant, only 12% of its total biomass is currently consumed. It can be utilized to produce value-added functional food via hydrolysis while enhancing its bioactivity and digestibility. Several recent studies have highlighted the antioxidant activities and anti-hypertensive properties of krill protein hydrolysates [9,12].

The current study is a continuation of our previous work, where the antioxidant activity of krill peptide hydrolysate (KPH) and its membrane fractions (<1 kDa, 1–3 kDa, and >3 kDa) were evaluated. The extraction was carried out using water as the medium. Due to the lower solubility of oil in water, no pretreatment was necessary to reduce oil contamination. Also, the aim of our current study is to develop functional food/food supplements, therefore, we omitted the use of any organic solution that would cause possible contamination per the requirements related to food safety and health. The extraction yield of KPH was 68.4%. The results showed that the 1–3 kDa fraction exhibited the highest antioxidant activities [9]. The amino acid composition of KPH and its fractions indicated a higher content of glutamic acid, aspartic acid, leucine, lysine, and arginine. In particular, the 1–3 kDa fraction had the highest content of aromatic amino acids when compared to KPH and other fractions. Based on previous studies, aromatic amino acids are considered effective radical scavengers and antioxidants. The higher content of aromatic amino acids in the 1–3 kDa fraction may have contributed to its comparatively higher antioxidant activity. Based on the above observations, a hypothesis was developed that KPF (1–3 kDa fraction) may possess protective effects against alcohol-induced oxidative liver damage in BALB/c mice; this was evaluated in the present study.

In ethanol-induced rats, food and water consumption seemed to decrease during the study period. Compared with the ethanol group, the body weight and survival rate of the KPF (50 and 100 mg/kg mice) group increased significantly. This result indicates that KPF has a protective effect against alcohol-induced toxicity. Ethanol-induced hepatic damage is characterized by hepatic marker enzymes such as AST and ALT. The elevation of these enzymes in serum suggests hepatic impairment, and they are commonly used to measure the extent of liver damage [13]. Also, it has been reported that heavy consumption of alcohol induces the production of cholesterol, which aggravates alcoholic liver disease [14]. Alcohol exaggerates the generation of ROS through hepatic cytochrome P450 2E1, resulting in oxidative stress and cell death. ROS-induced hepatic lipid peroxidation and hepatic cell death elevate hepatic enzymes levels in serum [10,15]. The present results indicate that AST, ALT, and total cholesterol levels in the liver significantly increased in the ethanol-treated group compared to the control group. However, the level of AST, ALT, and total cholesterol in the KPF-treated group significantly decreased in a dose-dependent manner. These results highlight the hepatoprotective effect of KPF, which could possibly be due to antioxidant properties. However, further analysis is needed to confirm the contribution of its antioxidant activities.

Lipid peroxidation occurs due to the rapid reaction of membrane lipids with ROS, which is generated during ethanol metabolism. It is involved in the pathogenesis of various liver diseases, including liver fibrogenesis leading to cirrhosis. MDA is a commonly used indicator of lipid peroxidation. MDA is a reactive aldehyde formed during the last stages of lipid peroxidation of polyunsaturated fatty acids in the plasma membrane. It is an indication of the extent of lipid peroxidation in organisms, which is indirectly related to the degree of cell degeneration [10]. The increased MDA level in liver homogenates of the ethanol-treated group was significantly decreased upon the application of KPF, suggesting its potential to ameliorate lipid-peroxidation induced hepatocyte damage.

ROS may result in cell death, which may proceed via apoptosis necrosis or autophagy. Apoptosis is the most common cellular suicide mechanism seen during differentiation, metamorphosis, and physiological cell turnover. It is mediated by a complex network of signaling pathways. Unlike necrosis, apoptosis causes minimal damage to neighboring tissues with harmful debris being cleared up by phagocytic cells, thus averting the risk of inflammation. Two of the main signaling pathways of apoptosis are considered to be mitochondria-mediated and death receptor-mediated pathways.

Investigations have been implemented to evaluate the effect of KPF on the expression of apoptosis-related factors, such as cleaved caspase-3, Bcl-2, and Bax. Bcl-2 family molecular mediators include pro-apoptotic (Bax, Bad, Bid) and anti-apoptotic (Bcl-2, Bcl-xL, Mcl-1) proteins. Among these proteins, Bax induces mitochondrial cytochrome c release and pro-apoptotic factors by disrupting voltage-dependent anion channels. Hence, Bax production is considered a key indicator of apoptosis [16]. Based on western blot analysis of apoptotic protein levels, alcohol administration increased the Bax level while decreasing the Bcl-2 level. Dose-dependent KPF application increased Bcl-2 expression and decreased expression of Bax, which indicates apoptosis prevention. Thus, KPF administration offers protective effects against alcohol-induced liver damage via regulating Bax/Bcl-2 expression and caspase-3 activation.

Alcohol exposure is reported to impair intracellular antioxidant systems mediated by enzymes such as SOD, CAT, and GPx that protect hepatocytes against oxidative damage [17]. Alcohol causes excessive ROS generation through hepatic cytochrome P450 2E1 activity, which is associated with a high level of NADPH oxidase activity, leading to the production of superoxide anion radicals and hydrogen peroxide in an agitated manner [10,18]. SOD catalytically increases the conversion of superoxide anion radicals to hydrogen peroxide, which is correlated with antioxidant effects and pathologic changes in the alcohol model of experimental ALD [7]. CAT is mainly localized in peroxisomes and readily reacts with hydrogen peroxide to form water and oxygen. GPx reduces hydrogen peroxide to water and lipid hydroperoxides. The above analysis suggests that KPF may have a desirable effect on ameliorating alcohol-induced oxidative stress.

Alcohol induces the release of inflammatory cytokines such as TNF- α and IL-6 in the liver, intensifying hepatic inflammation and apoptosis [19,20]. Both ROS and bacterial endotoxins leaking from the damaged intestine cause an inflammatory response during alcohol exposure. TNF- α and IL-6 are widely considered to be the most critical inflammatory cytokines in alcohol-induced liver damage [21]. Oxidative stress and inflammation are closely interrelated in alcohol-induced liver damage as inflammatory cytokines promote ROS generation and vice versa, and mitochondrial GSH depletion enhances liver sensitization to alcohol through TNF-α medicated hepatocellular death [22,23,24]. Based on the present evaluation, KPF markedly reduces hepatic production of both oxidative and inflammatory factors in alcohol-exposed mice. KPF could protect the liver through multiple paths.

Nrf2 is a transcription factor that drives a variety of downstream genes encoding detoxification enzymes and antioxidant proteins in response to oxidative stress. Nrf2 is physically attached to its specific repressor, Keap1, in the cytoplasm, which inhibits its actions. In response to oxidative stress stimuli, Nrf2 dissociates from Keap1, subsequently translocating into the nucleus, and combines with transcription factors. Then, the complex binds to the ARE and promotes the transcription of antioxidant genes, including HO-1, Nqo1 (NADPH oxidoreductase 1), and glutamate-cysteine ligase, which catalyzes the rate-limiting step in GSH synthesis [25,26,27]. Recently, Nrf2 has been recognized as a target for the treatment of ALD [28]. Additionally, HO-1 acts as a potent antioxidant with anti-inflammatory and anti-apoptotic functions and also improves cell survival in the liver. These findings suggest that the hepatoprotective effects of KPF against ethanol-induced liver damage might be attributed to its ability to reduce oxidative stress by enhancing antioxidant defense systems via the activation of the Nrf2/HO-1 pathway.

The positive control, silymarin, is a natural compound derived from Silybum marianum, which is commonly known as milk thistle. Silymarin is renowned for having hepatoprotective and antioxidant activities that act against free radicals produced during the metabolism of toxic substances such as ethanol and carbon tetrachloride. Silymarin is widely popular as a complementary alternative medicine for its pharmacological effects associated with the treatment of hepatic diseases. Therefore, silymarin is widely used as a positive control. Hepatic histopathological changes were taken into account in order to examine the alcohol-induced liver damage. The appearance of slight micro-vesicular steatosis, hepatocellular ballooning, and vacuole formation may vary due to alcohol intoxication, whereas the sightings of these were gradually reduced with dose-dependent treatment of KPF, similar to the liver tissues in mouse treated with silymarin. Fatty liver (hepatic steatosis) is observed during the initial stage of ALD and is characterized by hepatocyte triglyceride (TG) accumulation, which is assumed to reversible. However, hepatic steatosis is regarded as a potentially pathologic condition. With continuous alcohol consumption, hepatic steatosis may progress to advanced stages of ALD, resulting in steatohepatitis, cirrhosis, fibrosis, and possibly hepatocellular carcinoma [17]. The present findings imply that KPF offers hepatoprotective effects against alcohol-induced hepatic steatosis.

Our results suggest that krill protein hydrolysates possess hepatoprotective effects against alcohol-induced liver damage, and thus, hydrolysates and bioactive peptides prepared from krill can be employed as functional foods to enhance liver functions and health. Further studies could focus on evaluating the anti-inflammatory potential of KPF using nuclear factor κB (NF-κB) pathway molecular mediators, which supposedly drive the major hepatic inflammatory reactions in Kupffer cells [17].

## 4. Materials and Methods

### 4.1. Materials

Krill (*Euphausia superba*) was purchased from Dongwon Co. (Busan, Korea) and stored at –20 °C. Pepsin was obtained from Junsei Chemical Co. (Tokyo, Japan). Enzyme assay commercial kits were purchased from Biovision (Milpitas, CA, USA). Silymarin, Thiobarbituric acid was acquired from Sigma-Aldrich (St Louis, MO, USA). Glutamic oxaloacetic transaminase (AST) and glutamic pyruvic transaminase (ALT) enzymatic analysis kit were purchased from Asan Pharmaceuticals. (Hwasung, Korea). Regents and primers for PCR were purchased from Invitrogen, Gaithersburg, MD, USA, and Promega, Madison, WI, USA. All other used reagents were of the highest purity grade.

### 4.2. Preparation of Peptide Fraction from KPH by Gastrointestinal Digestion

Krill protein hydrolysate was prepared according to the previously described method by using pepsin according to the previously described method [9]. Briefly, krill dry powder and pepsin at an enzyme/substrate ratio of 1:100 (*w/w*) were mixed, adjusted to pH 2, and then hydrolysis was carried out under continuous agitation at 37 °C for eight hours. The mixture was then heated in a 100 °C water bath for 10 min to heat-inactivate pepsin. Un-hydrolyzed proteins were removed by filtering through a filter cloth, and the filtrate was fractionated using ultrafiltration membranes to obtain three fractions: <1 kDa, 1–3 kDa, and >3 kDa using a Quixstand bench-top system (GE Healthcare, Buckinghamshire, UK). The 1–3 kDa peptide fraction (KPF) was lyophilized and used for further studies.

### 4.3. Animals

Male Balb/c mice (6-week-old, 23–26 g body weight) were obtained from Dae Han Bio Link CO. LTD (Eumseong, Korea). The animals were housed adhering to the Guidelines for Care and Use of Laboratory Animals of Chonnam National University. The animals were acclimated to temperature (22 ± 2 °C) and humidity-controlled (55 ± 5%) rooms with a 12 h light/dark cycle for one week before the experiments. The animals were fed tap water and a standard chow diet, which contain 15% protein, 50% polysaccharides, 7% simple sugars, 3% fat (*w/w*) energy 3.5 kcal/g. All mice procedures were approved by the Institutional Animal Care and Use Committee of Chonnam National University (No. CNU IACUC-YS-2016-5).

### 4.4. Experimental Design

Balb/c mice were randomly divided into five groups (*n* = 6) with treatment once a day (09:00–10:00) for 10 days: control (saline), ethanol (ethanol 3 g/kg mice), KPF50 (ethanol 3 g/kg + KPF 50 mg/kg mice), KPF100 (ethanol 3 g/kg + KPF 100 mg/kg mice), and silymarin (ethanol 3 g/kg + silymarin 50 mg/kg mice). During the experimental period, the body weights and survival rates were monitored daily (08:00–09:00). The mice were anesthetized after ten days, and blood samples were collected into heparin tubes to determine biochemical parameters. Livers were immediately frozen by liquid nitrogen and stored at –70 °C until analysis. Livers were homogenized in five volumes of ice-cold homogenization buffer (250 mM sucrose, 50 mM Tris-HCl, pH 7.4, 1 mM EDTA). The liver homogenates were centrifuged at 1000× *g* for 10 min, and the supernatants were centrifuged at 12,000× *g* for 30 min. The final supernatant protein concentration was measured by the Bradford method using bovine serum albumin as the protein standard [29]. The experimental protocols were approved by the Animal Ethics Committee of Chonnam National University.

### 4.5. Serum Biochemical Analysis

The activity of glutamic oxaloacetic transaminase (AST) and glutamic pyruvic transaminase (ALT) in the plasma samples was determined using an enzymatic analysis kit (Asan Pharmaceuticals, Hwasung, Korea), according to the Reitmen-Frankel method [30]. The total cholesterol content in serum was determined using a commercial kit (Asan Pharmaceuticals, Hwasung, Korea) according to the method in [31].

### 4.6. Determination of Lipid Peroxidation

Lipid peroxidation concentrations in serum and liver homogenates were determined by measuring thiobarbituric acid reactive substances (TBARS) based on the method in [32]. Briefly, serum or liver homogenates were mixed with thiobarbituric acid (TBA) and incubated in boiling water for 30 min. After centrifugation at 1000× *g* for 10 min, the resulting colored upper layer was measured at 532 nm. The TBARS concentration was expressed as nmol of malondialdehyde (MDA) per ml serum or mg protein.

### 4.7. Determination of Antioxidant Enzyme Activity in the Liver

Antioxidant enzyme activity for superoxide dismutase (SOD), catalase (CAT), and glutathione peroxidase (GPx) in liver homogenates was determined using commercially available kits (Biovision, Milpitas, CA, USA) following the manufacturer’s instructions.

### 4.8. Reverse Transcription-Polymerase Chain Reaction (RT-PCR) Analysis

Total RNA was isolated from liver using a Trizol reagent (Invitrogen, Gaithersburg, MD, USA) according to the manufacturer’s instructions. For cDNA synthesis, 2 μg total RNA was mixed with reverse transcription system (Promega, Madison, WI, USA). The target genes are designed by the program of gene-specific primers, and the target cDNA was amplified using the following primers: forward 5′-CCA CAG CTG AGA GGG AAA TC-3’ and revers 5′-TGG GGG ACA GCT TCC TTC TT-3′ for TNF-α; forward 5’-TTC CAG AAT CCC TGG ACA AG-3’ and reverse 5’-CAG AAT TGC CAT TGC ACA AC-3’ for IL-6; forward 5′-TCA-CCT-GGA-AGA-CAG-CTC-CT-3′ and reverse 5’-AAG GAA GGC TGG AAA AGA GC-3’ for β-actin. The PCR amplification was carried out with 35 cycles as follows: denaturation at 95 °C for 45 s, annealing at 60 °C for 1 min, and extension at 72 °C for 45 s. After 35 cycles, the PCR products were separated by electrophoresis on 1% agarose gel for 30 min at 100 V and visualized with UV light after ethidium bromide staining.

### 4.9. Western Blot Analysis

The liver homogenates were mixed with buffer (60 mM Tris-HCl, 2% SDS, and 2% β-mercaptoethanol, pH 7.2), and boiled for 5 min. Sample at 40–80 μg protein was applied to 10–12% sodium dodecyl sulfate-polyacrylamide gel (SDS-PAGE), electrophoresed, and transferred to a nitrocellulose membrane. After blocking with a solution containing 5% nonfat milk for 2 h to prevent non-specific binding of the antibody, segmented membrane strips were incubated with primary antibodies against NF-κB p50, NF-κB p60, ERK, JNK, p38, Bax, Bcl-2, cleaved caspase-3, and β-actin overnight at 4 °C followed by incubation for two hours with horseradish peroxidase-conjugated secondary antibodies (1:3000). Finally, protein bands were detected using an enhanced chemiluminescence western blotting detection kit (Pierce Biotechnology, Rockford, IL, USA). The bands were imaged on DavinchChemi imaging system (Core Bio, Seoul, Korea) [33].

### 4.10. Hematoxylin and Eosin Staining for Histological Analysis

Liver tissues were fixed in 10% (*v/v*) phosphate buffer formalin and embedded in paraffin wax. The paraffin sections (4–6 μm thick) were segmented, and each was stained using hematoxylin and eosin stain and examined under an optical microscope (Olympus DP70, Olympus Optical Co., Tokyo, Japan) integrated with a camera [34].

### 4.11. Statistical Analysis

The data from the animal experiments are presented as mean ± standard error (SE), and all statistical comparisons were carried out via one-way analysis of variance followed by Tukey’s test using PASW Statistics 19.0 software (SPSS, Chicago, IL, USA). A *p*-value < 0.05 was considered significant.

## Figures and Tables

**Table 1:** Effects of the KPH on hepatic biomarkers in alcohol-fed mice.

Group	AST (IU/L)	ALT (IU/L)	Total Cholesterol (mg/dL)
Normal	63.76 ± 1.95 ^a^	14.88 ± 0.56 ^a^	56.07 ± 1.49 ^a^
Ethanol	92.88 ± 4.96 ^d^	24.36 ± 1.21 ^d^	76.15 ± 5.15 ^c^
KPF50	85.08 ± 3.98 ^c^^,^^d^	21.00 ± 0.49 ^c^	67.25 ± 4.43 ^b^
KPF100	78.91 ± 2.56 ^b,c^	18.77 ± 0.70 ^b^	59.51 ± 1.80 ^a^
Silymarin	72.00 ± 7.30 ^a,b^	17.82 ± 0.80 ^b^	56.84 ± 3.00 ^a^

Abbreviations, AST; aspartate transaminase, ALT; alanine transaminase, and MDA; malondialdehyde. Values mean ± SE of three determinations (*n* = 6). Bars with different letters are significantly different (*p* < 0.05). ^a–d^ Values with different superscripted letters are significantly different (*p* < 0.05).

## References

[1] You Y., Yoo S., Yoon H.-G., Park J., Lee Y.-H., Kim S., Oh K.-T., Lee J., Cho H.-Y., Jun W. (2010). In vitro and in vivo hepatoprotective effects of the aqueous extract from Taraxacum officinale (dandelion) root against alcohol-induced oxidative stress. Food Chem. Toxicol..

[2] Amini M., Runyon B.A. (2010). Alcoholic hepatitis 2010: A clinician’s guide to diagnosis and therapy. World J. Gastroenterol. WJG.

[3] Kim S.-K., Ravichandran Y.D., Khan S.B., Kim Y.T. (2008). Prospective of the cosmeceuticals derived from marine organisms. Biotechnol. Bioprocess Eng..

[4] Das S.K., Vasudevan D. (2007). Alcohol-induced oxidative stress. Life Sci..

[5] Ambade A., Mandrekar P. (2012). Oxidative stress and inflammation: Essential partners in alcoholic liver disease. Int. J. Hepatol..

[6] Lu Y., Cederbaum A.I. (2008). CYP2E1 and oxidative liver injury by alcohol. Free Radical Biol. Med..

[7] Wu D., Cederbaum A.I. (2003). Alcohol, oxidative stress, and free radical damage. Alcohol Res. Health.

[8] Nikoo M., Benjakul S. (2015). Potential application of seafood-derived peptides as bifunctional ingredients, antioxidant–cryoprotectant: A review. J. Funct. Foods.

[9] Park S.Y., Je J.-Y., Ahn C.-B. (2016). Protein Hydrolysates and Ultrafiltration Fractions Obtained from Krill (Euphausia superba): Nutritional, Functional, Antioxidant, and ACE-Inhibitory Characterization. J. Aquat. Food Prod. Technol..

[10] Je J.-Y., Cha J.-Y., Cho Y.-S., Ahn H.-Y., Lee J.H., Cho Y.-S., Ahn C.-B. (2013). Hepatoprotective effect of peptic hydrolysate from salmon pectoral fin protein byproducts on ethanol-induced oxidative stress in Sprague–Dawley rats. Food Res. Int..

[11] Wang L., Xue C., Wang Y., Yang B. (2011). Extraction of Proteins with Low Fluoride Level from Antarctic Krill (Euphausia superba) and Their Composition Analysis. J. Agric. Food Chem..

[12] Hatanaka A., Miyahara H., Suzuki K.I., Sato S. (2009). Isolation and Identification of Antihypertensive Peptides from Antarctic Krill Tail Meat Hydrolysate. J. Food Sci..

[13] Kasdallah-Grissa A., Mornagui B., Aouani E., Hammami M., El May M., Gharbi N., Kamoun A., El-Fazaâ S. (2007). Resveratrol, a red wine polyphenol, attenuates ethanol-induced oxidative stress in rat liver. Life Sci..

[14] Ko S.-C., Kang N., Kim E.-A., Kang M.C., Lee S.-H., Kang S.-M., Lee J.-B., Jeon B.-T., Kim S.-K., Park S.-J. (2012). A novel angiotensin I-converting enzyme (ACE) inhibitory peptide from a marine Chlorella ellipsoidea and its antihypertensive effect in spontaneously hypertensive rats. Process Biochem..

[15] Weber L.W., Boll M., Stampfl A. (2003). Hepatotoxicity and mechanism of action of haloalkanes: Carbon tetrachloride as a toxicological model. Crit. Rev. Toxicol..

[16] Fernando I.P.S., Jayawardena T.U., Kim H.-S., Lee W.W., Vaas A.P.J.P., De Silva H.I.C., Abayaweera G.S., Nanayakkara C.M., Abeytunga D.T.U., Lee D.-S. (2019). Beijing urban particulate matter-induced injury and inflammation in human lung epithelial cells and the protective effects of fucosterol from Sargassum binderi (Sonder ex J. Agardh). Environ. Res..

[17] Ding R.-B., Tian K., He C.-W., Jiang Y., Wang Y.-T., Wan J.-B. (2012). Herbal medicines for the prevention of alcoholic liver disease: A review. J. Ethnopharmacol..

[18] Ingelman-Sundberg M., Johansson I., Yin H., Terelius Y., Eliasson E., Clot P., Albano E. (1993). Ethanol-inducible cytochrome P4502E1: Genetic polymorphism, regulation, and possible role in the etiology of alcohol-induced liver disease. Alcohol.

[19] Cohen J.I., Chen X., Nagy L.E. (2011). Redox signaling and the innate immune system in alcoholic liver disease. Antioxid. Redox Signal..

[20] Mandrekar P., Ambade A., Lim A., Szabo G., Catalano D. (2011). An essential role for monocyte chemoattractant protein-1 in alcoholic liver injury: Regulation of proinflammatory cytokines and hepatic steatosis in mice. Hepatology.

[21] Neuman M.G. (2003). Cytokines-central factors in alcoholic liver disease. Alcohol Res. Health.

[22] Fernandez-Checa J.C., Kaplowitz N. (2005). Hepatic mitochondrial glutathione: Transport and role in disease and toxicity. Toxicol. Appl. Pharmacol..

[23] Hoek J.B., Pastorino J.G. (2002). Ethanol, oxidative stress, and cytokine-induced liver cell injury. Alcohol.

[24] Yan S.-L., Yang H.-T., Lee H.-L., Yin M.-C. (2014). Protective effects of maslinic acid against alcohol-induced acute liver injury in mice. Food Chem. Toxicol..

[25] Cao Y.-W., Jiang Y., Zhang D.-Y., Wang M., Chen W.-S., Su H., Wang Y.-T., Wan J.-B. (2015). Protective effects of Penthorum chinense Pursh against chronic ethanol-induced liver injury in mice. J. Ethnopharmacol..

[26] Lamlé J., Marhenke S., Borlak J., Von Wasielewski R., Eriksson C.P., Geffers R., Manns M.P., Yamamoto M., Vogel A. (2008). Nuclear Factor-Eythroid 2–Related Factor 2 Prevents Alcohol-Induced Fulminant Liver Injury. Gastroenterology.

[27] Wu K.C., Liu J., Klaassen C.D. (2012). Role of Nrf2 in preventing ethanol-induced oxidative stress and lipid accumulation. Toxicol. Appl. Pharmacol..

[28] Bataille A., Manautou J. (2012). Nrf2: A potential target for new therapeutics in liver disease. Clin. Pharmacol. Ther..

[29] Bradford M.M. (1976). A rapid and sensitive method for the quantitation of microgram quantities of protein utilizing the principle of protein-dye binding. Anal. Biochem..

[30] Reitman S., Frankel S. (1957). A colorimetric method for the determination of serum glutamic oxalacetic and glutamic pyruvic transaminases. Am. J. Clin. Pathol..

[31] Allain C.C., Poon L.S., Chan C.S., Richmond W., Fu P.C. (1974). Enzymatic determination of total serum cholesterol. Clin. Chem..

[32] Ohkawa H., Ohishi N., Yagi K. (1979). Assay for lipid peroxides in animal tissues by thiobarbituric acid reaction. Anal. Biochem..

[33] Fernando I.P.S., Sanjeewa K.K.A., Samarakoon K.W., Lee W.W., Kim H.-S., Kang N., Ranasinghe P., Lee H.-S., Jeon Y.-J. (2017). A fucoidan fraction purified from Chnoospora minima; a potential inhibitor of LPS-induced inflammatory responses. Int. J. Biol. Macromol..

[34] Kim K.-N., Kang M.-C., Kang N., Kim S.-Y., Hyun C.-G., Roh S.W., Ko E.-Y., Cho K., Jung W.-K., Ahn G. (2015). 2,4,6-Trihydroxybenzaldehyde, a potential anti-obesity treatment, suppressed adipocyte differentiation in 3T3-L1 cells and fat accumulation induced by high-fat diet in C57BL/6 mice. Environ. Toxicol. Pharmacol..

